# Degenerative joint disease induced by repeated intra-articular injections of monosodium urate crystals in rats as investigated by translational imaging

**DOI:** 10.1038/s41598-021-04125-7

**Published:** 2022-01-07

**Authors:** Nathalie Accart, Janet Dawson, Michael Obrecht, Christian Lambert, Manuela Flueckiger, Julie Kreider, Shinji Hatakeyama, Peter J. Richards, Nicolau Beckmann

**Affiliations:** 1 Musculoskeletal Diseases Department, Novartis Institutes for BioMedical Research, Fabrikstr. 28.3.04, CH-4056 Basel, Switzerland; 2 Autoimmunity, Transplantation & Inflammation Department, Novartis Institutes for BioMedical Research, Lichtstr. 35, WSJ-386.6.08.18, CH-4056 Basel, Switzerland

**Keywords:** Imaging, Biomarkers, Diagnostic markers, Experimental models of disease, Preclinical research, Translational research, Bone, Cartilage, Muscle

## Abstract

The objective of this work was to assess the consequences of repeated intra-articular injection of monosodium urate (MSU) crystals with inflammasome priming by lipopolysaccharide (LPS) in order to simulate recurrent bouts of gout in rats. Translational imaging was applied to simultaneously detect and quantify injury in different areas of the knee joint. MSU/LPS induced joint swelling, synovial membrane thickening, fibrosis of the infrapatellar fat pad, tidemark breaching, and cartilage invasion by inflammatory cells. A higher sensitivity to mechanical stimulus was detected in paws of limbs receiving MSU/LPS compared to saline-injected limbs. In MSU/LPS-challenged joints, magnetic resonance imaging (MRI) revealed increased synovial fluid volume in the posterior region of the joint, alterations in the infrapatellar fat pad reflecting a progressive decrease of fat volume and fibrosis formation, and a significant increase in the relaxation time T_2_ in femoral cartilage, consistent with a reduction of proteoglycan content. MRI also showed cyst formation in the tibia, femur remodeling, and T_2_ reductions in extensor muscles consistent with fibrosis development. Repeated intra-articular MSU/LPS injections in the rat knee joint induced pathology in multiple tissues and may be a useful means to investigate the relationship between urate crystal deposition and the development of degenerative joint disease.

## Introduction

Gout represents a highly prevalent form of arthritis, characterized by recurrent episodes of painful acute inflammatory flares in response to monosodium urate (MSU) crystals that deposit predominantly in peripheral joints and surrounding tissues^1^. Long-term deposition of MSU crystals can result in joint damage. Moreover, gout and osteoarthritis (OA) often occur concomitantly, and a positive correlation between synovial fluid uric acid and OA has been established^2^. Nevertheless, it is presently not known whether and how these conditions are pathologically linked^3–5^.

Recent advances in the understanding of crystal-induced inflammation provided further support for a shared inflammatory pathway between gout and OA. MSU crystals activate the macrophage innate immune response via the Nacht Domain, leucine-rich repeat, and pyrin domain-containing protein 3 (NALP3) inflammasome, which is required for caspase-1 activation and subsequent interleukin (IL)-1β and IL-18 release^6^. The implication of IL-1β in the pathogenesis of gout has been confirmed by the successful treatment of patients with severe gout using IL-1β inhibition^7^. In in vitro studies, either the Toll-like receptor 4 (TLR-4) agonist lipopolysaccharide (LPS) or phorbol myristate acetate had to be used to prime cells for effective MSU crystal-induced IL-1β release^6,8^. Also in the clinics, TLR signaling and priming of inflammasome have been described as a critical step in the process of gouty flares^9,10^. IL-1β is considered to play a central role in OA as well^11,12^, and synovial fluid levels of both IL-1β and IL-18 are strongly associated with OA severity^2^.

Preclinical models play an important role in facilitating research in this area. Previous studies demonstrated that a single intra-articular injection of MSU crystals resulted in gouty-arthritis in mice and rats^13,14^. However, these studies were limited to the acute phase of the response, with an observation time of up to 2 days only, following crystal administration.

In this work, we investigated the long-term effect of repeated injections of MSU crystals in combination with lipopolysaccharide (LPS) into the knee joints of rats every two weeks for a maximum of five administrations, with the aim to stimulate NLRP3 inflammasome activation as previously described^6,15^. We assessed the swelling response after each injection, paw withdrawal sensitivity to mechanical stimuli using electric von Frey, as well as hard and soft tissue joint changes by magnetic resonance imaging (MRI) and micro-computed tomography (micro-CT). Histology was performed at selected time points for the characterization of pathological features at the cellular level. The objective was to assess the consequences of repeated intra-articular crystal/LPS administration with inflammasome priming in order to simulate recurrent gout bouts in rats, using translational imaging to simultaneously detect and quantify injury in different areas of the knee joint.

## Materials and methods

### Statement on animal welfare

In vivo experimental procedures followed the Swiss animal welfare regulations. The experimental protocols were approved by the Cantonal Veterinary Office of the City of Basel, Switzerland. The study was performed under the license number BS-1438, approved by the Cantonal Veterinary Office of the City of Basel. Authors complied with the ARRIVE guidelines for animal experimentation.

### Animals

Female Lewis/OrlRj rats (n = 25) from Janvier Laboratories (Le Genest-Saint-Isle, France), 150–180 g or eight weeks of age at the beginning of the study, were used. Rats were housed under standard conditions (12-h light/dark cycle), with standard chow and water provided ad libitum. Upon arrival, rats were allowed two weeks of acclimatization before beginning the experiments.

### Monosodium urate (MSU) crystals

MSU crystals were prepared according to the method reported by Reber et al.^16^. Initially, 1.0 g of uric acid was added to 6.0 mL of 1.0 N NaOH and 194 mL of double distilled water and then, the mixture was heated. The pH of this mixture was adjusted to 7.2 with 1.0 N HCl. The mixture was allowed to cool slowly at room temperature and then stored at 4 °C for 24 h. Finally, the monourate crystals were dried after washing and then, the crystals were suspended in sterile saline (100 mg/ml).

### Induction of gouty arthritis

Twenty five rats were used. On day 0, the right knee received 50 µl of a mixture containing 40 mg/ml MSU crystals and 0.1 mg/ml LPS from E. coli (0111:B4, Sigma L2630) in saline, while the left knee of the same animal received 50 µl of saline. The intra-articular injections of MSU/LPS into the right and saline into the left knee were then repeated every two weeks (namely on days 0, 14, 28, 42 and 56). Saline was administered into the left knee to verify whether repeated intra-articular injection of fluid might elicit an inflammatory response in the knee joint. Rats were anesthetized with 3.5% isoflurane (Abbott, Cham, Switzerland)/air for each intra-articular injection. At each of the time points days 14, 28, 42, 56 and 70 five animals were culled for post-mortem analyses. The sacrificed rats had received the last MSU/LPS dose 14 days before being euthanized.

A preliminary dose–response was performed to select the MSU and LPS dose which led to a robust swelling and cytokine detection in the synovial fluid 24 h after intra-articular dosing (supplementary Fig. 1). The choices for the doses of MSU and LPS for the dose–response were based on published literature (Marcotti et al^13^ for MSU, De-Melo et al^17^ and Ahmad et al^18^ for LPS).

### Exclusion criterion

A weight loss of more than 20% would lead to an exclusion and early euthanasia of an animal. However, no rat needed to be excluded from the study.

### Knee swelling

Knee diameters were measured using calipers immediately before and again on days 1, 2, 3 and 7 after each intra-articular injection. Right and left knee diameters were determined in the medial–lateral direction, with the caliper positioned perpendicularly to the leg axis. Knee swelling was defined as the ratio between the knee diameter at a given time point and the mean knee diameter at baseline, before any injection.

### Nociceptive test

Hind-paw sensitivity was evaluated by measuring the mechanical withdrawal threshold using a handheld electronic von Frey unit (Cat # 38450, Ugo Basile, Gemonio, Italy). An animal was placed in a clear box on an elevated mesh screen (models BIO-STD EVF and BIO-PVF, Bioseb, Vitrolles, France), and allowed to habituate for 15 min before testing. A filament was applied to the plantar surface of each hind paw. The force was increased by increments of 0.1 g force units from zero until paw withdrawal. A transducer comprising a digital timer automatically recorded the force eliciting paw withdrawal and the corresponding response latency to the nearest 0.1 s. The filament was applied five times per paw, separated by a 5-min interval to prevent sensitization, and the threshold was defined as an average of the five withdrawals observed within the trials.

### Imaging

During acquisitions animals were anesthetized with isoflurane 1.5–2% in air, administered via a nose cone.

#### MRI

Performed with a Pharmascan 7 Tesla scanner (Bruker, Ettlingen, Germany). A T_2_-weighted spin-echo sequence with the following parameters was applied: 16 echoes spaced by 11 ms, echo time (TE) from 11 to 176 ms, repetition time 2022 ms, pixel size 0.078 × 0.078 mm, slice thickness 0.48 mm, 8 slices, without and with fat suppression. A volume resonator (Model 1P-T11070V3, Bruker) with 72 mm inner diameter was used for transmission. A two-channel phased array receive-only mouse head surface coil (Model 1P-T11204V3, Bruker) was used for signal reception. Joints receiving MSU/LPS were imaged at all the time points specified. In contrast, joints receiving saline were measured solely at baseline and at day 69.

Relaxation time T_2_ for cartilage, infrapatellar pad and muscle was determined by fitting with GraphPad Prism (version 8.1.2, GraphPad Software, San Diego, CA) the corresponding signals from regions-of-interest (ROIs) placed in these anatomical areas as function of TE. Volumes of effusion were determined by segmenting the corresponding signals by their intensity using a region grower algorithm available at the scanner software.

#### Micro-CT

Measurements were performed using a vivaCT-40 micro-CT system (Scanco Medical, Brüttisellen, Switzerland). The scan parameters were: voxel size 17.5 × 17.5x17.5 μm, 426 slices, integration time 130 ms, high resolution, 55 E(kVp), 145 μA, 8 W mode, cone beam continuous rotation.

### Ex vivo analyses

Two weeks after one or more MSU/LPS injections, rats were euthanized, and synovial fluid collected from the right and left knees. To recover synovial fluid from the joints, the skin was cut vertically to expose the knee joint. The tissue over the knee was carefully opened using scissors and forceps, and the surrounding tissue and patella tendon over the knee was held to form a pocket over the center of the knee. Saline (20 µl) was injected into the knee joint, followed by massaging of the joint to help distribute the saline and free-up synovial fluid from knee compartments. The capsule was then opened, and lavaged a further two times with saline (20 µl). The fluid within the joint was then extracted using a needle and syringe, centrifuged at 2000×*g* for 10 min at 4 °C, and the supernatant stored at − 80 °C until further analysis. The skin was removed and knees were excised for histology.

### Multiplex ELISA

Synovial fluid samples were analyzed using a multiplex enzyme-linked immunosorbent assay (ELISA) with rat specific reagents, following the manufacturer’s protocols (Bio-Rad Laboratories, Hercules, CA). Cytokines tested were interleukin-1α (IL-1α), IL-1β, keratinocyte chemoattractant/growth-regulated oncogene (KC/GRO), macrophage inflammatory protein-1α (MIP-1α), monocyte chemoattractant protein-1 (MCP-1), and vascular endothelial growth factor (VEGF). The dedicated Bio-Plex Manager™ software running on a Bioplex 200 System array reader (Bio-Rad) was used to determine individual concentrations. Because of the small amount of synovial fluid drawn from each animal, data from different time points during the course of the study were pooled to allow statistical comparisons between saline- and MSU/LPS-injected joints.

### Histological preparation

Knee joints were fixed in 10% neutral buffered formalin for three days and then placed in a decalcification solution (ImmunoCal Cat # 1440, Decal Chemical Corp, Suffern, NY) for 5 days. On the fourth day, knees were trimmed along the sagittal axis approximately at the mid-trochlear level to separate medial and lateral condyles of the joint, and decalcification was pursued until completion. After sample dehydration and paraffin embedding, 5-µm-thick sections were cut on the lateral condyle and stained: hematoxylin and eosin (H&E) for the characterization of synovium and proteoglycan-containing cartilage identified by Safranin O/Fast green using a procedure adapted from Lillie and Fulmer^19^ and from Prophet et al^20^. Macrophages and osteoclasts were detected with an anti-CD68 antibody (MCA341R, Serotec, Puchheim, Germany) applied on paraffin sections as described by Damoiseaux et al^21^.

### Analyses of histological changes

Histological assessment was performed on the patello-femoral cartilage (PFC), the anterior femoral cartilage (AFC) and the anterior tibial cartilage (ATC). Joint pathology was scored according to the Mankin system^22^, considering cartilage surface integrity (0–6), proteoglycan loss (0–4), chondrocyte morphology (0–3), fibrovascular replacement of subchondral marrow fat spaces (0–1), synovitis (0–3) and tidemark breaching (0–1). This methodology was adapted from Takahashi et al^23,24^. All histological scores were determined by one blinded and trained observer (N.A.).

### Statistics

Multiplex assay data from synovial fluid samples were analyzed using Student’s *t*-tests (Origin 2021, OriginLab Corporation, Northampton, MA, USA). Paw withdrawal threshold and MRI data were analyzed using ANOVA with random effects (Systat version 13; Systat Software Inc., San Jose, California, USA) to take into account the longitudinal structure of the data. Tukey post-hoc tests were performed for specific comparisons between groups. Mankin scores were analyzed using Mann–Whitney tests (Origin 2021). Pearson correlation was determined between T_2_ in the infrapatellar fat pad and the latency time in the von Frey assessments. A value of p < 0.05 was considered statistically significant.

## Results

Repeated injection of MSU/LPS every two weeks into the knee joint for a maximum of 5 times induced marked swelling of the injected joint, joint inflammation, synovial membrane thickening, fibrosis of the infrapatellar fat pad, proliferation of the synovial membrane, breaching of the tidemark and cartilage invasion by inflammatory cells. Principal actors of the MSU/LPS-induced response were neutrophils, mast cells and macrophages (supplementary Fig. 2). All these features are present in human gout pathology^25^.

### Knee swelling and inflammatory markers in synovial fluid

**Figure 1:**
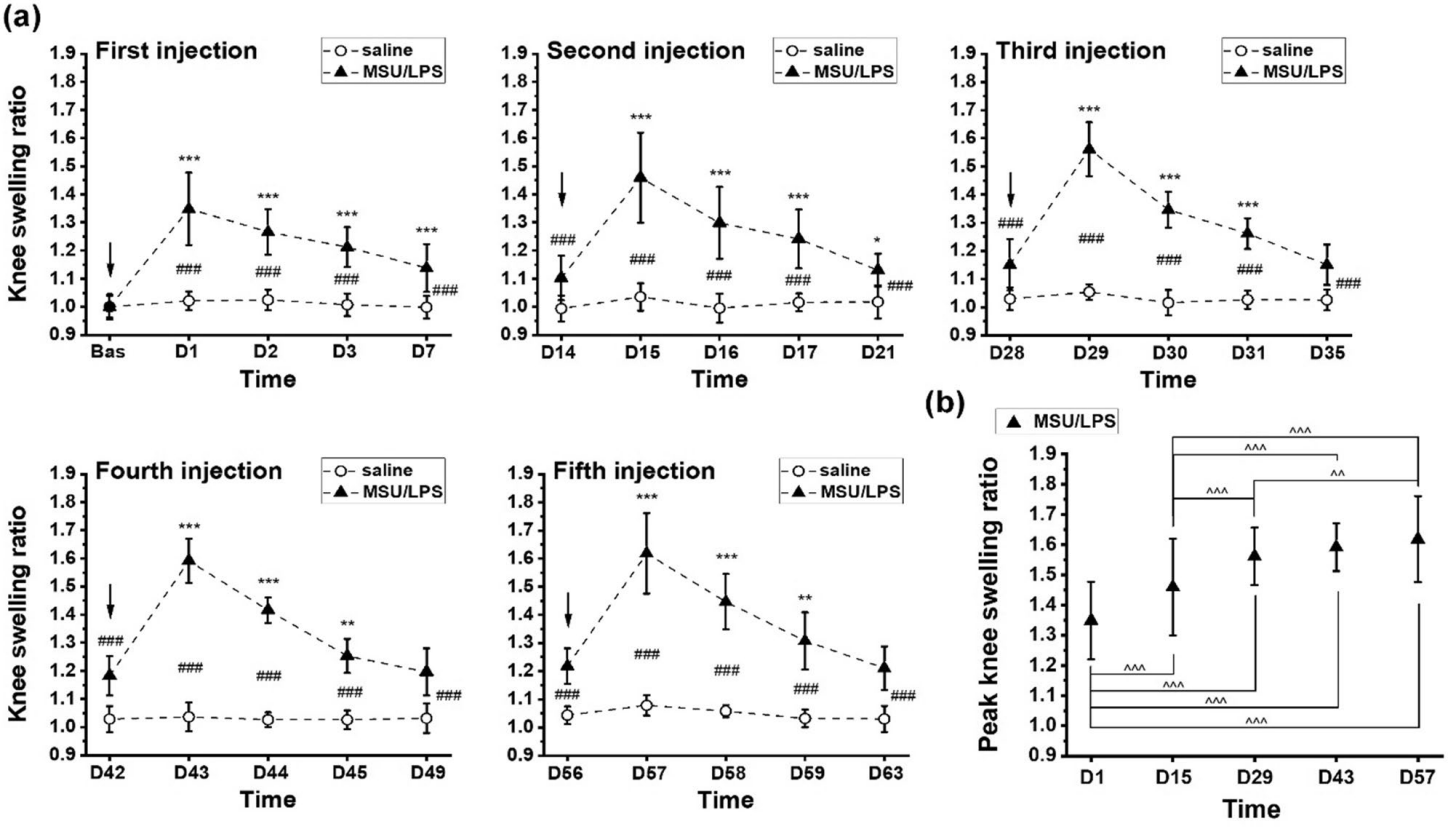
Knee swelling ratio. (**a**) Data for every injection of saline or MSU/LPS. Arrows indicate the injection time points. (**b**) Peak knee swelling ratios (at one day after every injection) for MSU/LPS-challenged joints. Values are expressed as mean ± sd. ANOVA with random effects statistics were performed for all swelling data shown. Significance levels: ### *p* < 0.001 for comparisons between saline and MSU/LPS-treated joints; within the MSU/LPS group: *0.01 < *p* < 0.05, **0.001 < *p* < 0.01, ***p < 0.001 for comparisons of values just before with values up to day 7, for every injection; ^^0.001 < *p* < 0.01, ^^^*p* < 0.001 for comparisons of peak swelling ratios.

Intra-articular administration of saline led to a small increase in left knee diameter compared to baseline, reaching a maximum of 7% after the fifth injection (Fig. 1a). Swelling was maximal at day 1 after each MSU/LPS injection (Fig. 1a), attaining joint diameter increase of 35% and 62% from the first to the fifth challenge. Knees remained slightly swollen just before the next administration of crystals, and this slight additional swelling increased up to the fourth challenge. The maximum (peak) swelling increased after each injection until the third MSU/LPS administration (Fig. 1b). Subsequent injections did not increase the maximum knee swelling response further.

**Figure 2:**
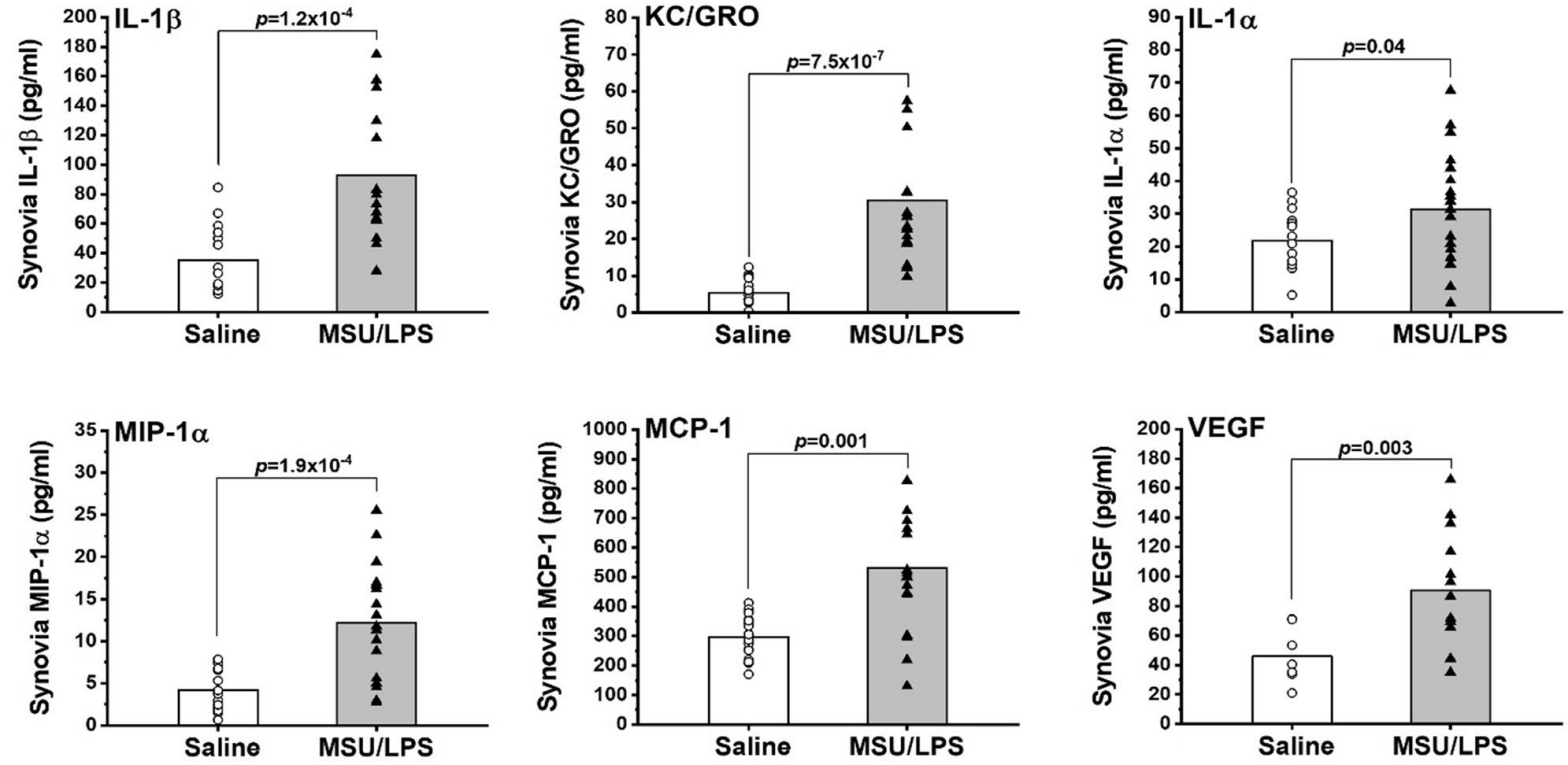
Inflammatory markers in the synovial fluid of joints injected with saline or MSU/LPS. Data from several time points during the course of the study have been pooled and presented as means (vertical bars) or individually. The significance levels refer to *t*-test comparisons between left and right joints, injected with saline and MSU/LPS, respectively.

Figure 2 summarizes a number of markers in the synovial fluid that were increased in MSU/LPS- compared to saline-injected joints. Because of the limited sample volume per joint at each individual time point, data were pooled for the statistical analysis. The levels of IL-1α, IL-1β, KC/GRO (the IL-8 related protein in rodents^26^), MIP-1a, MCP-1 and VEGF were higher in the synovial fluid of MSU/LPS-treated joints.

### Paw sensitivity to mechanical stimulus

**Figure 3:**
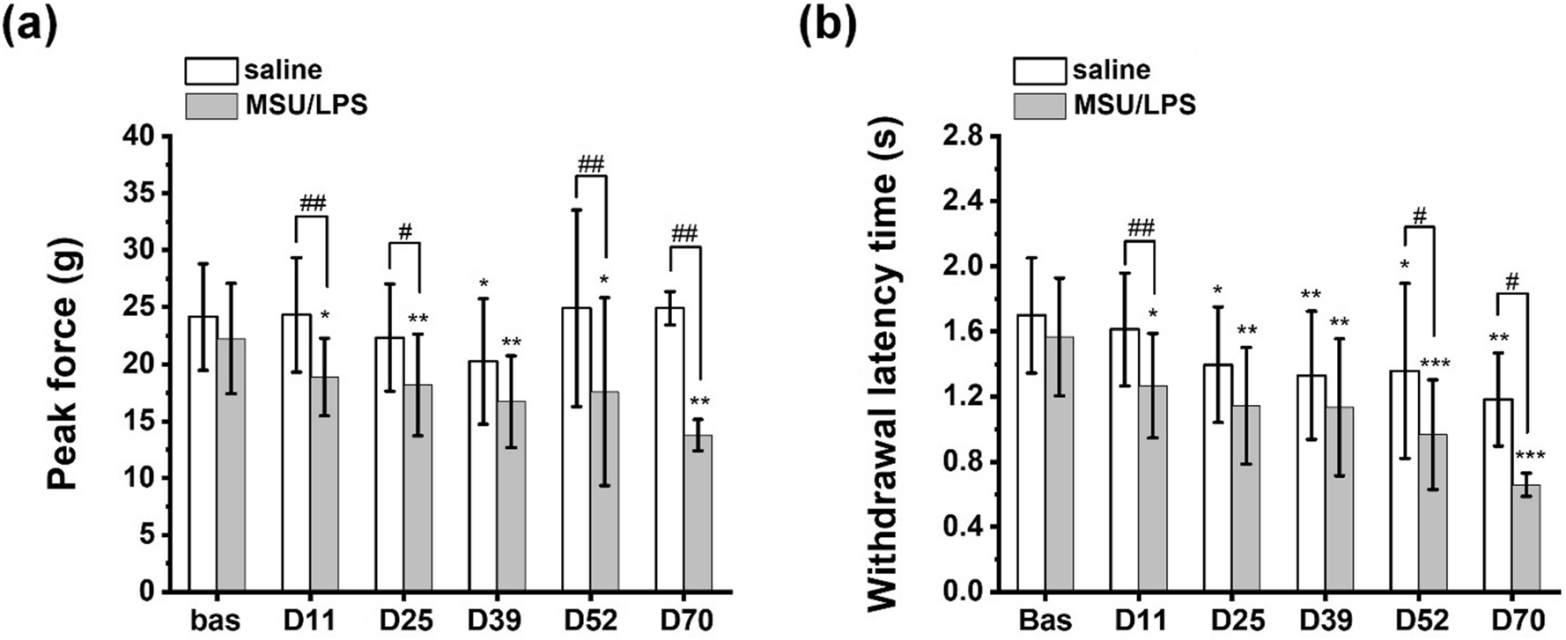
Nociceptive test involving stimulation of the plantar region of hind paws by electric von Frey. (**a**) Peak force and (**b**) withdrawal latency time upon stimulation are provided as mean ± sd. Data were analyzed by ANOVA with random effects. The significance levels * 0.01 < *p* < 0.01, ** 0.001 < *p* < 0.01, *** p < 0.001 refer to comparisons to baseline values in the same group; # 0.01 < *p* < 0.05, ## 0.001 < *p* < 0.01 refer to comparisons between left and right hind paws, comprising saline- and MSU/LPS-injected knees, respectively.

The nociceptive test revealed that the peak force applied for left hind limb withdrawal was unchanged during the course of the study. By contrast, the peak force applied for right hind limb withdrawal was decreased from the first injection of MSU/LPS onwards (Fig. 3a). Moreover, at several time points the withdrawal latency was significantly shorter in MSU/LPS-treated right hind limbs as compared to saline-treated left hind limbs (Fig. 3b). These data indicated a higher paw sensitivity to mechanical stimulus on the right side, corresponding to the knee which received the injections of MSU crystals.

### In vivo imaging and histology

**Figure 4:**
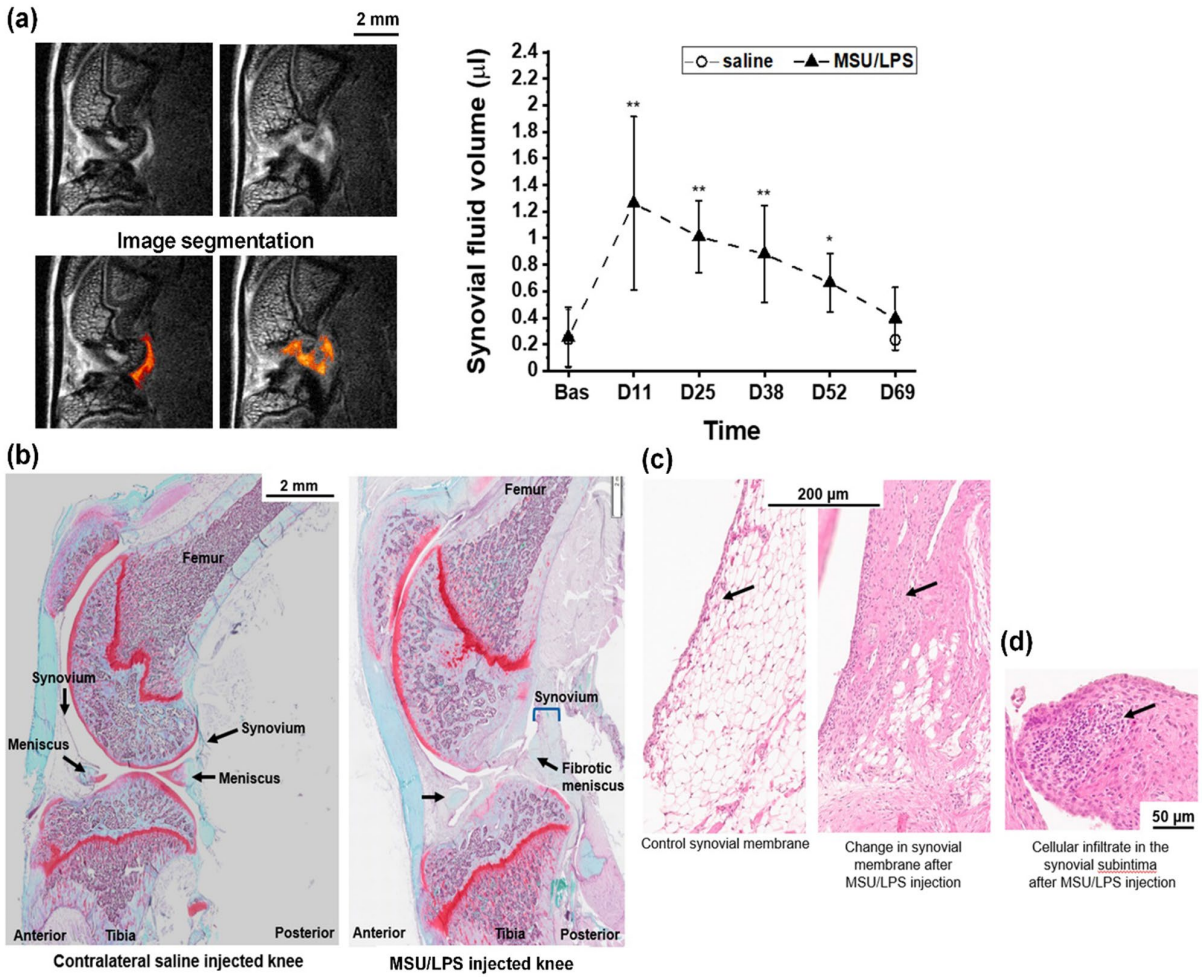
Synovial effusion in MSU/LPS-injected knee joints. (**a**) Two MRI images acquired from the knee joint at day 11 after crystal administration (upper row). Corresponding segmentation of the synovial region (lower row) to illustrate the assessment of synovial fluid volume summarized in the graph. Values denote means ± sd. The significance levels * 0.01 < *p* < 0.05, ** 0.001 < *p* < 0.01 correspond to ANOVA with random effects comparisons to baseline values. (**b**) Sagittal histological section of knee joint following two injections of saline (left control) or MSU crystals/LPS (right). Both histology pictures were obtained from Safranin O-stained sections. (**c**) Histology at day 14 after a single injection of MSU/LPS showing thickening of the lining of the synovial membrane and the cellular density increase in the sublining. (**d**) Histology of the inflammatory infiltration in the sublining of the synovial membrane at day 70. All histology pictures in (**c**,**d**) were obtained from Hematoxylin/Eosin-stained sections.

A significant and sustained increase of the synovial fluid volume in the posterior joint region was determined by MRI in joints treated with MSU/LPS (Fig. 4a). No inflammation was observed in the synovial membrane from a saline-injected joint (Fig. 4b). In contrast, synovial inflammation was confirmed by histology, which identified a higher cellular density within the thickened membrane 14 days after a single MSU/LPS injection (Fig. 4c). This feature was accentuated after further MSU/LPS injections until lymphoid infiltration in the synovial subintimal layer (Fig. 4d).

**Figure 5:**
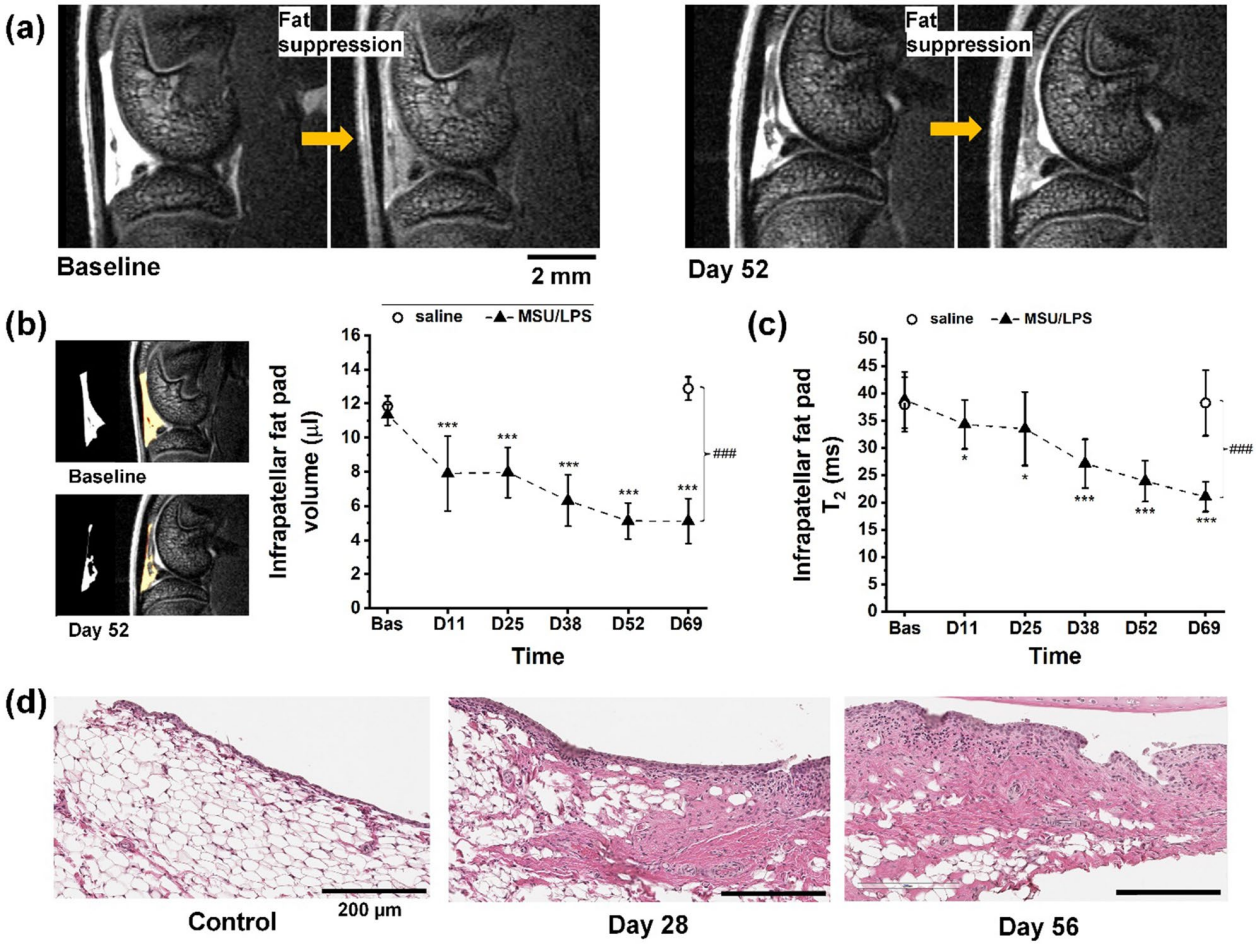
Changes in the infrapatellar fat pad induced by MSU/LPS. (**a**) Representative MRI images from the joint of the same animal at baseline and at day 52 (day 10 after the fourth injection of MSU crystals), acquired without and with fat suppression. (**b**) Fat pad volume (mean ± sd) obtained by segmenting the corresponding fat signal from non-suppressed images. (**c**) Fat pad T_2_ (mean ± sd) assessed in fat-suppressed images. Significance levels * 0.01 < *p* < 0.05, ***p < 0.001 correspond to ANOVA with random effects comparisons to baseline values, ^###^p < 0.001. (**d**) Histology of MSU/LPS-injected joints (mid and right panels) illustrating the influx of inflammatory cells, fat reduction and fibrosis formation in the infrapatellar fat pad. For comparison, fat pad of a saline-injected joint (left panel). All histology images were obtained from Hematoxylin/Eosin-stained sections.

In the Hoffa’s infrapatellar fat pad area, non-fat suppressed MRI revealed a significant decrease of fat volume starting at day 11 after the first MSU/LPS injection (Fig. 5a,b). Additional analyses of fat-suppressed images showed a significant T_2_ reduction over the experimental time (Fig. 5c). At day 69 (day 13 after the fifth administration of crystals), T_2_ in the infrapatellar fat pad (21.0 ± 2.7 ms) was significantly lower by 46% (*p* = 0.0001) compared to baseline (38.8 ± 5.2 ms). Moreover, it was significantly lower by 45% (*p* = 0.0006) with respect to infrapatellar fat pad T_2_ values of saline-injected joints at the same time point (38.2 ± 6.0 ms). The T_2_ decrease in the infrapatellar fat pad was consistent with fat reduction and/or extracellular matrix remodeling (fibrosis) revealed by histology. Two weeks after the second MSU/LPS injection, numerous cells had already invaded the infrapatellar fat pad (Fig. 5d). On day 52, i.e. 10 days after the fourth injection of crystals, histology demonstrated significant fat reduction and fibrosis in this region (Fig. 5d). A positive correlation was found between T_2_ in the infrapatellar fat pad and the latency time in the von Frey assessments (R = 0.43, p = 0.005).

**Figure 6:**
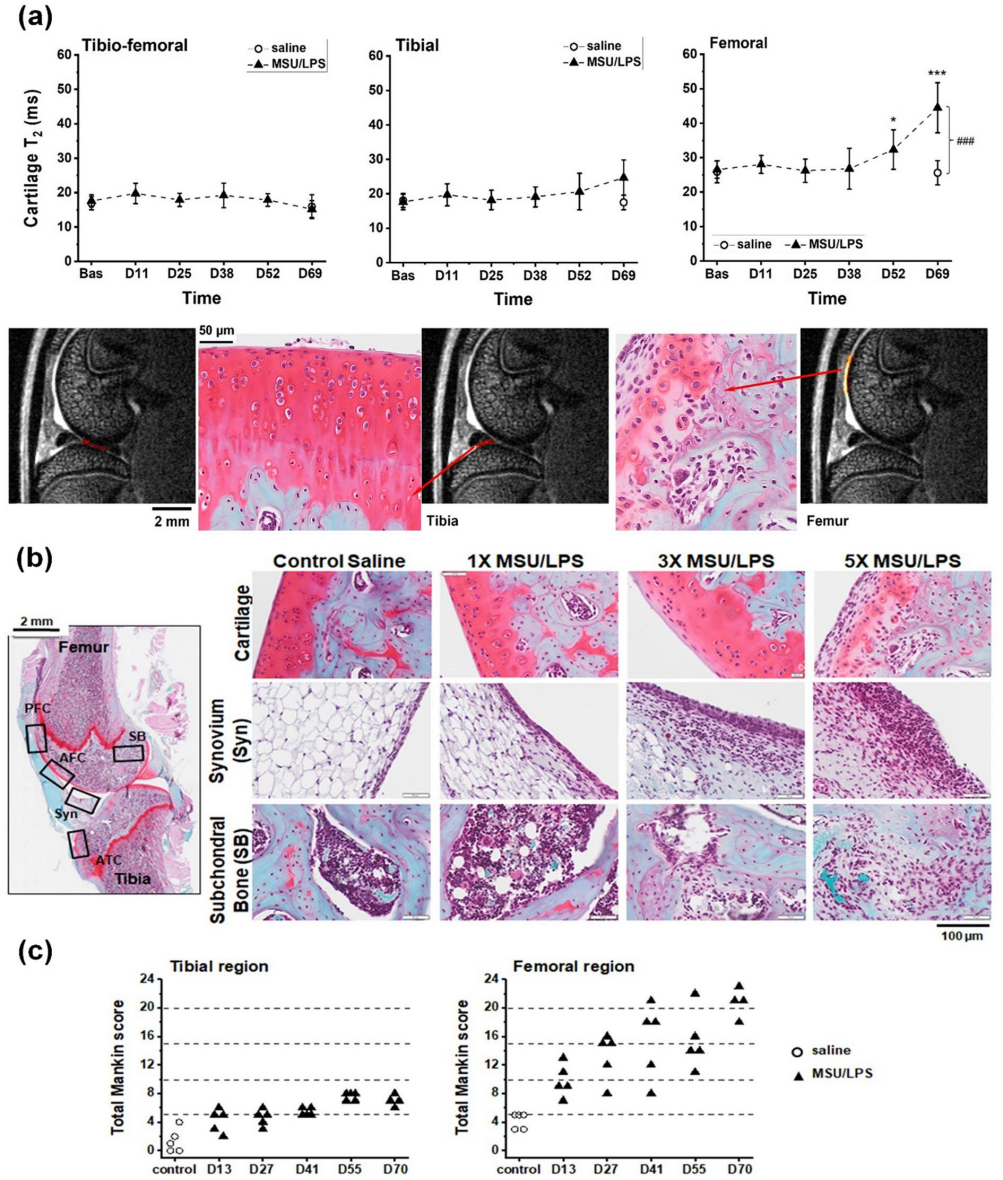
Changes in cartilage induced by MSU/LPS. (**a**) Relaxation time T_2_ (mean ± sd) assessed in different cartilage areas as shown on an MRI image acquired at day 69 (day 13 after the fifth administration of MSU/LPS). A significant increase in T_2_ during the experimental period was observed in femoral cartilage, consistent with histology at day 70 demonstrating cartilage remodeling in the same area. In contrast, T_2_ of tibial cartilage remained practically unchanged. The significance levels * *p* = 0.03 and *** *p* < 0.001 relate to ANOVA with random effects comparisons to baseline values, ^###^p < 0.001. T_2_ was assessed on fat-suppressed images. (**b**) Representative histological images to illustrate pathological features in several joint areas in the model. AFC anterior femoral cartilage; ATC anterior tibial cartilage; PFC patellar femoral cartilage; SB subchondral bone; Syn synovium. (**c**) Total Mankin score for the anterior tibial and femoral regions of saline- or MSU/LPS-challenged joints. Scores were given according to the criteria cartilage surface integrity (0–6), proteoglycan loss (0–4), chondrocyte morphology (0–3), fibrovascular replacement of subchondral marrow fat spaces (0–1), synovitis (0–3) and tidemark breaching (0–1), resulting in a maximum score of 18. For the femoral region, Mankin scores from the patello-femoral cartilage and the anterior femoral cartilage were added. Controls corresponded to contralateral joints after five saline injections.

Starting at day 52, MRI additionally revealed a significant increase in cartilage T_2_ in the medial femoral condyle with respect to baseline values, whereas tibial cartilage T_2_ values remained constant (Fig. 6a). At day 69, the femoral cartilage T_2_ of joints receiving MSU/LPS (44.5 ± 7.3 ms) was significantly increased by 68% (p = 0.0002) with respect to baseline (26.5 ± 2.5 ms). It was also significantly increased by 74% (p = 0.001) with respect to T_2_ values in saline-injected contralateral joints (25.6 ± 3.5 ms). The T_2_ increase in femoral cartilage was consistent with a reduction in proteoglycan staining detected by histology predominantly in front of the patella (Fig. 6a). Histology of cartilage also revealed fibrillation and detachment of the superficial layer, an increased number of chondrocyte clusters and the presence of hypertrophic chondrocytes in the middle zone (Fig. 6b). Moreover, we observed tidemark breaching and cellular infiltration in the cartilage associated to the presence of osteoclasts. Cartilage OA Mankin scores and representative histological images are presented in Fig. 6b,c. Consistent with the MRI results, Mankin scores for MSU/LPS-challenged joints were significantly higher in femoral compared to tibial regions (p < 0.05). Moreover, a progression of the damage was detected by histology with the number of MSU/LPS administrations. Mankin scores for saline-challenged joints were low.

**Figure 7:**
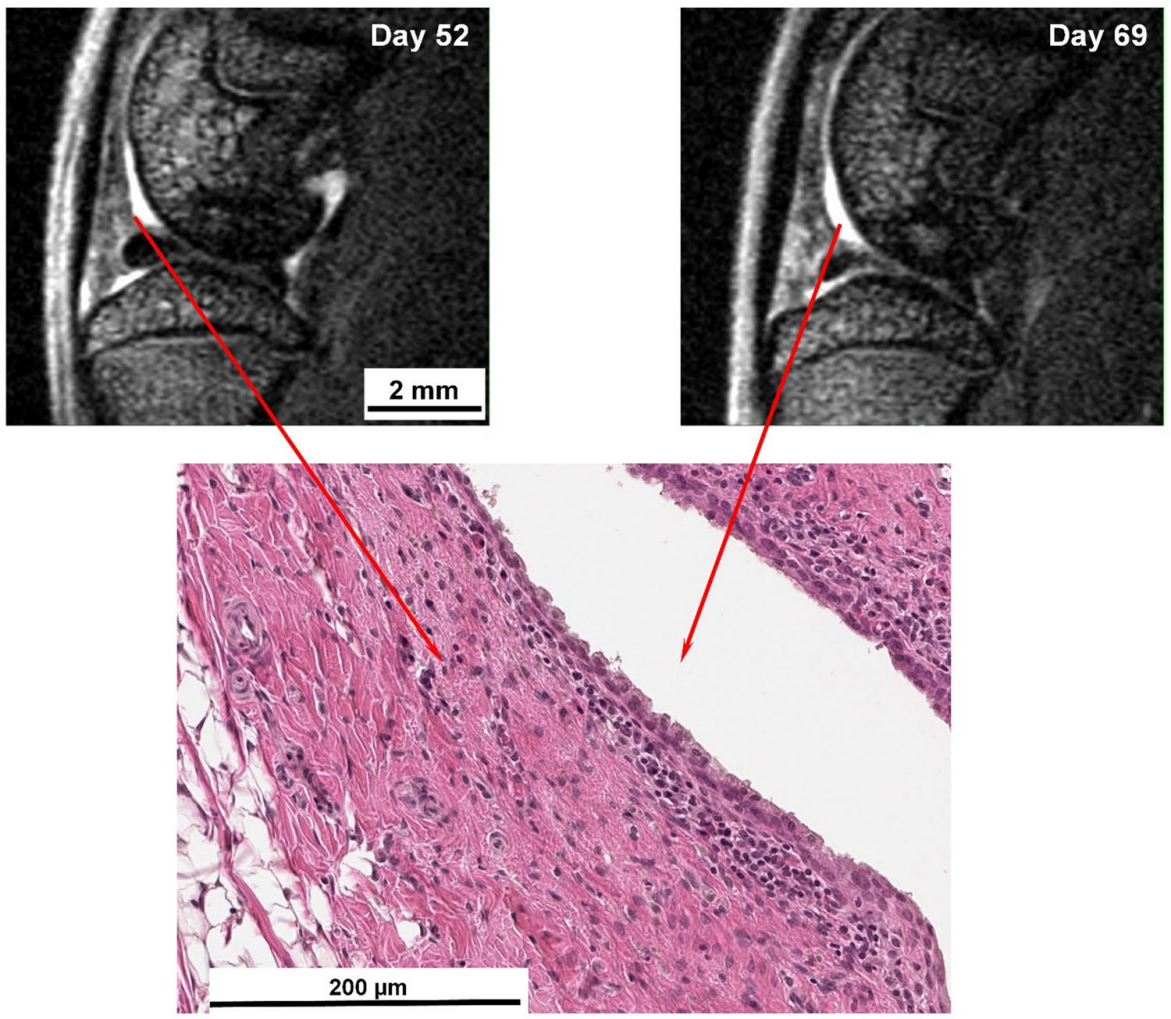
Hypertrophy of the synovial membrane induced by MSU/LPS. MRI images of the knee joint acquired from two rats at days 52 (day 10 after the fourth crystals administration) and 69 (day 13 after the fifth crystals injection). Histological analysis from the same joints demonstrated cell density increase in the synovial membrane and thickening by cellular infiltration (Hematoxylin/Eosin-stained section). T_2_ values in this region were of about 70 ms.

Synovial membrane thickening and proliferation were detected by histology at days 52 and 69 (day 10 after the fourth and day 13 after the fifth crystal administration, respectively) in the anterior portion of the joint (Fig. 7). Macrophage proliferation was observed in the hypertrophic synovial membrane (supplementary Fig. 2). These findings might explain the large increase in T_2_ observed in and around the femoral cartilage close to the meniscus (Fig. 7).

**Figure 8:**
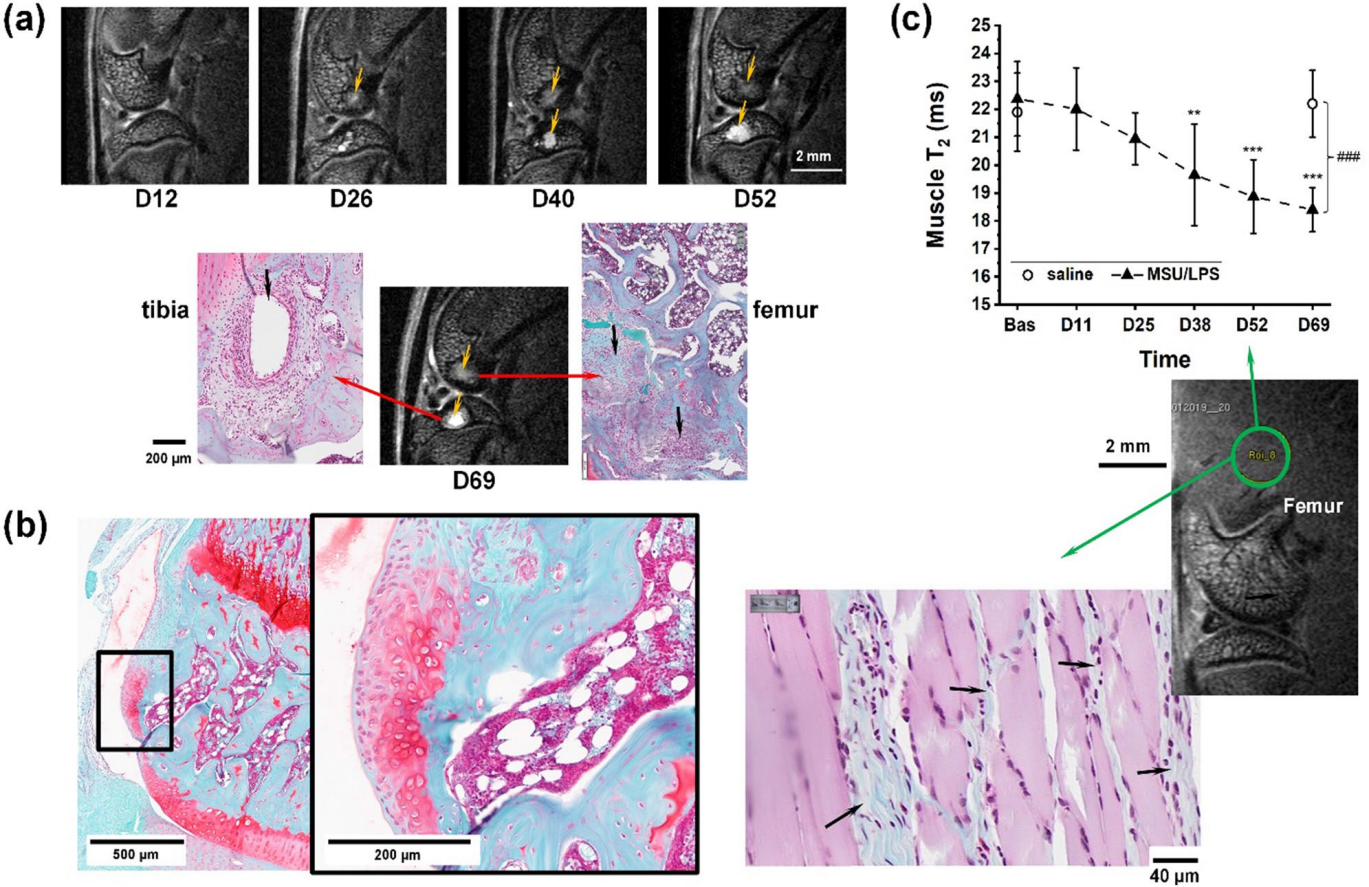
Changes in bone and muscle induced by MSU/LPS. (**a**) MRI images acquired longitudinally from the knee joint of the same rat. Histology at day 69 revealed that the contrast change in MRI starting to appear at day 26 on the femur was consistent with bone remodeling. Moreover, the structure highlighted on the tibia from day 40 was a cyst. (**b**) Histology at day 70 showed the formation of chondrophytes in the tibia. (**c**) The decreased in relaxation time T_2_ (mean ± sd) in the knee extensor muscles was consistent with the development of fibrosis as evidenced by histology. The significance levels **p = 0.001 and ***p < 0.001 relate to ANOVA with random effects comparisons to baseline values, ^###^p < 0.001. T_2_ was assessed on fat-suppressed images.

Changes in bone structure were also observed. Fifty percent of the rats injected with MSU/LPS presented with cyst formation in the tibia, and bone remodeling in the femur, with the development of a woven bone structure as determined by MRI and histology (Fig. 8a). Moreover, chondrophytes appeared at the margins of cartilage, predominantly in the medial proximal condyle of the tibia, as seen in all coronal sections of animals injected four or five times with MSU/LPS (Fig. 8b). These chondrophytes, which were not detectable by micro-CT (supplementary Fig. 3), formed in the periosteum at the junction between cartilage and bone. Finally, a reduction of T_2_ was detected in a region comprising the knee extensor muscles (Fig. 8c). This reduction was consistent with fibrosis revealed by histology in the same area.

## Discussion

In the present study, we utilized longitudinal and endpoint measurements to characterize a rat model of urate crystal-induced joint injury. Our findings demonstrate that an early osteoarthritic-like phenotype can be generated following multiple intra-articular challenges with MSU/LPS, thereby lending further support to the concept of a link between gout and OA.

Mast cells, macrophages and polymorphonuclear cells are considered the main drivers of urate crystal-induced arthritis^25^. We were able to observe polymorphonuclear cells appearing in the bone marrow and to detect tissue invasion by mast cells and macrophages in rat knee joints during the early time points following MSU/LPS challenge. Similarly, we also observed increases in several soluble pro-inflammatory proteins. IL-1β synovial fluid levels were upregulated, with a significant increase reported overall. This was expected, as IL-1β is produced as a direct consequence of inflammasome activation with MSU crystals following LPS priming^6^. The characteristic influx of neutrophils and macrophages during a gout flare is coordinated by cytokines/chemokines including IL-1β, IL-8 (CXCL8), MCP-1 (CCL2) and MIP1 (CCL3)^27,28^. Upregulation of these mediators was observed in our model following intra-articular challenge with MSU/LPS. Synovial inflammation with cell influx and proliferation is also associated with the production of new blood vessels via the process of angiogenesis. Hypoxia within the proliferating synovial tissue contributes to this process with the production of vascular endothelial growth factor (VEGF), mainly in rheumatoid arthritis, but also in OA joint disease^29^. VEGF was increased in the synovial fluid from all MSU/LPS injected joints at each of the five MSU injection time points, suggesting that angiogenesis was induced throughout the development of this gouty arthritis model. This inflammatory reaction further translated into knee swelling peaking in the days immediately after the MSU/LPS injection. The peak swelling ratio increased up to the third MSU/LPS injection, and remained constant following subsequent challenges. This was suggestive of a habituation of the biological system to the inflammatory stimuli elicited by the crystals, in agreement with other inflammatory models. For instance, repeated ovalbumin administration in rats was found to reduce its capacity to induce lung inflammation^30^. Further evaluation using non-invasive MRI identified synovial effusion within MSU/LPS-treated joints, peaking at day 11.

Cartilage damage is often observed in joints affected by advanced gout^31^. Chhana et al^32^ demonstrated that MSU crystals contribute to cartilage damage in gout through a reduction in chondrocyte viability and function, and increased catabolic activity within cartilage. Here, MSU/LPS challenge also led to biochemical and structural changes in articular cartilage, as evidenced by MRI and histology. The collagen-proteoglycan matrix limits the mobility of water protons, thus promoting relaxation, leading to low T_2_’s in hyaline cartilage. At baseline, cartilage T_2_ (26.5 ± 1.5 and 17.6 ± 1.6 ms for femoral and tibial cartilage, respectively) was in good agreement with literature values measured at 7 Tesla in healthy humans (24.9 ± 1.3 ms for femoral and 19.2 ± 1.0 ms for tibial cartilage)^33^ and rats^34,35^. Because of its sensitivity to changes in collagen content and orientation^36^, as well as in proteoglycan content^37^, MRI T_2_ mapping can identify abnormalities of the cartilage extracellular matrix and probe early stages of cartilage degeneration occurring prior to macroscopic cartilage defects and thinning. Increase in cartilage T_2_ relaxation time in the absence of erosion thus reflects proteoglycan depletion and/or changes in the collagen organization^38–40^. Here, T_2_ increased primarily in femoral cartilage, close to the patellar region, by 21% and 68% compared to baseline at days 52 and 69, respectively. Histology revealed proteoglycan reduction predominantly in the femoral cartilage at these time points, in areas displaying increased T_2_. Other rat models of cartilage injury showed comparable T_2_ increases. For instance, an increase by approximately 40% and 50% was detected three weeks after meniscectomy^41^ or anterior cruciate ligament transection^35^. Immobilization of the knee joint for two weeks led to cartilage T_2_ increase by approximately 60%^34^. The regional distribution of crystal-induced cartilage degeneration was consistent with earlier work demonstrating proteoglycan degradation predominantly in the anterior femoral cartilage in front of the patella following intra-articular injection of papain^42^ or other chemicals^43^. Finally, proliferation of the synovial membrane with a large increase in relaxation time T_2_ was also detected in the present model. Synovitis comprising the influx of inflammatory cells (lymphocytes, macrophages, neutrophils) and the proliferation of fibroblast-like synoviocytes, forming pannus and leading to cartilage and ultimately bone destruction, is an important feature of gout^2^ and OA^44^.

Along with cartilage, menisci, ligaments, synovium and synovial fluid, fat pads are major constituents of the knee joint. The infrapatellar Hoffa’s pad, one of the largest fat pads, plays a role in facilitating the distribution of synovial fluid and mechanical forces throughout the joint. This fat pad, which produces several pro-inflammatory adipokines and cytokines, such as, TNF-α, IL-6, and leptin, as well as VEGF, can influence inflammatory processes in the knee^45^. Hoffa’s disease is characterized by inflammation, hypertrophy, and fibrosis of the pad in response to repetitive trauma^46^. Moreover, the infrapatellar fat pad and synovial membranes in OA were shown to be more inflamed, vascularized and fibrous compared with those of healthy individuals^47^. Here, MRI revealed significant decrease of the relaxation time T_2_ at the level of the infrapatellar fat pad during the course of the experiment. Since water molecules interacting with collagen and other macromolecules have very short T_2_’s^48^, collagen production and tissue remodeling are expected to lead to a reduction in T_2_. The decreased T_2_ observed here was thus consistent with fat decrease and fibrosis development revealed by histology. In clinics, a shortening of T_2_ was reported for the infrapatellar fat pad chronically after arthroscopy surgery indicating tissue fibrosis^49^. Moreover, larger infrapatellar fat pad volume has been associated with greater knee cartilage volume and fewer structural abnormalities, suggesting a protective role of infrapatellar fat pad size in knee OA^50^. The reduction in fat pad volume detected by MRI and confirmed by histology would therefore be in accordance with the development of relevant knee joint pathology in our model.

The infrapatellar fat pad is richly innervated^51^, being one source of anterior knee pain^52^. Either acute or chronic trauma can lead to local bleeding, inflammation and eventually to fibrotic lesions, all of which are causes of infrapatellar fat pad-related pain^46^. Preclinically, irreversible structural changes in the infrapatellar fat pad, such as extensive fibrosis, were described to occur prior to persistent pain development in Wistar rats receiving a single intra-articular injection of monoiodoacetic acid^53^. In our study, a positive correlation was found between the T_2_ values of the infrapatellar fat pad in fat-suppressed images and the withdrawal latency time of the paw upon von Frey filament stimulation. Despite this positive correlation, a more detailed investigation would be necessary to verify whether T_2_ assessments of the infrapatellar fat pad could serve as an objective surrogate measure of increased paw sensitivity in the present crystal-induced knee joint injury model. Of note, a significant decrease of infrapatellar fat pad T_2_ was detected at day 11 in fat-suppressed images only, suggesting that changes in fat pad at this early time point were primarily driven by cell invasion/tissue remodeling as confirmed by histology. At later time points, T_2_ reductions in non-suppressed and fat-suppressed images were consistent with fat reduction and tissue remodeling/fibrotic lesion development evidenced histologically. Concerning the nociceptive test, while the withdrawal latency was significantly shorter in the MSU-crystal-injected leg, there was also a shorter withdrawal latency in the contralateral leg. Since the animals were growing, the higher sensitivity could be a natural weight compensation. More weight balanced on the non-injured leg might have affected the sensitivity in this leg. In order to properly evaluate this possibility, a naive control group without knee injections would be required in future experiments. Another potential explanation for the contralateral leg having a shorter withdrawal latency is the chronic, centralized pain in the model.

Changes in knee loading are considered to be an important contributor to the development of knee OA in humans. It has been found that the quadriceps muscle, which plays a central role in modulating loads across the knee joint, may be up to 40% weaker in OA patients than in healthy individuals^54^. Such weakness contributes not only to pain and loss of function in knee OA, but is observed early in the disease process, often preceding disease onset^54^. Lower quadriceps function in moderate OA has been associated with extracellular matrix (ECM) expansion in muscle^55^. In addition, higher profibrotic gene expression^55^ and fibrosis^56^ were found in the quadriceps and the vastus medialis muscle in patients with knee OA. Here, we observed a reduction of the T_2_ relaxation time in muscle, consistent with fibrosis observed by histology. Of note, a trend towards reduced T_2_ appeared on day 25 and the effect became highly significant at later time points, while femoral cartilage damage became apparent at days 53 and 69 only. These results suggest that ECM remodeling in muscle described for knee OA patients was recapitulated in the present animal model. Although not carried out here, non-invasive assessment of muscle atrophy as described by Giorgetti et al^57^ will be performed in future studies.

Cartilage outgrowths, or chondrophytes, were observed in our model. These undergo endochondral ossification during gout^58^ or OA progression^59^. However, we did not detect osteophytes per se neither by micro-CT nor by histology. An increased study duration might have provided the opportunity for the detection of osteophytes.

Another shortcoming of the present study is the fact that young female rats were used, although gout is more prevalent in 30–60-year-old men and in post-menopausal women^60^. Repeating the study in male and/or older rats of different strains would be of interest. Moreover, it would be interesting as well to verify the response for several MSU crystal doses and sizes. Also, the omission of LPS, or the inclusion of other common NLRP3 priming agents, might provide further insights into the role of the inflammasome in this model.

Taken together, our findings indicate that the repeated injection of MSU crystals, in combination with LPS, into the knee joints of rats every two weeks leads to distinctive pathological changes reflecting degenerative joint disease. This model provides the opportunity to simulate repeated bouts of gouts in a small rodent, and to study possible consequences of them. The changes detected were multifactorial, with cartilage and bone alterations coexisting with pathological features of inflammation and hyperplasia. Finally, the results reported here serve to illustrate the power of non-invasive translational imaging to longitudinally quantify pathology in multiple tissues of the knee joint.

## Supplementary Information

Supplementary Information.

## Abbreviations

AFC: Anterior femoral cartilage
ATC: Anterior tibial cartilage
CT: Computed tomography
ECM: Extracellular matrix
ELISA: Enzyme-linked immunosorbent assay
KC/GRO: Keratinocyte chemoattractant/growth-regulated oncogene (IL-8)
IL-1β: Interleukin-1β
IL-18: Interleukin-18
LPS: Lipopolysaccharide
MCP-1: Monocyte chemoattractant protein-1
micro-CT: Micro-computed tomography
MIP-1α: Macrophage inflammatory protein-1α
MRI: Magnetic resonance imaging
MSU: Monosodium urate
NLRP3: NACHT, LRR and pyrin domains-containing protein 3
OA: Osteoarthritis
PFC: Patello-femoral cartilage
ROI: Region-of-interest
sd: Standard deviation
sem: Standard error of the means
SYN: Synovium
TE: Echo time
TR: Repetition time
VEGF: Vascular endothelial growth factor
