# Supplementary material for: Degenerative joint disease induced by repeated intra-articular injections of monosodium urate crystals in rats as investigated by translational imaging
Source: Sci Rep. 2022 Jan 7;12:157. doi: 10.1038/s41598-021-04125-7 (PMC8742129; doi:10.1038/s41598-021-04125-7)
Supplement: Supplementary file 1 — Supplementary Information. [file 41598_2021_4125_MOESM1_ESM.docx]

**Degenerative joint disease induced by repeated intra-articular injections of monosodium urate crystals in rats as investigated by translational imaging**

**Nathalie Accart^1,*^, Janet Dawson^2,*^, Michael Obrecht^1^, Christian Lambert^1^,**

**Manuela Flueckiger^1^, Julie Kreider^1^, Shinji Hatakeyama^1^, Peter Richards^1^, Nicolau Beckmann^1,#^**

**^1^Musculoskeletal Diseases Department and ^2^Autoimmunity, Transplantation ＆ Inflammation Department, Novartis Institutes for BioMedical Research, CH-4056 Basel, Switzerland**

*Contributed equally to this work.

#Corresponding author: PD Dr. Nicolau Beckmann

Novartis Institutes for BioMedical Research

Musculoskeletal Diseases Department

Fabrikstr. 28.3.04, CH-4056 Basel, Switzerland

E-mail: [nicolau.beckmann@novartis.com](mailto:nicolau.beckmann@novartis.com)


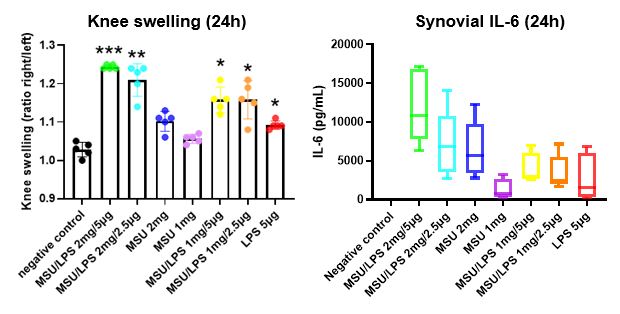


**Supplementary figure 1 –** Initial dose finding for the MSU/LPS combination. The doses of MSU and LPS were chosen based on knee swelling and IL-6 content in synovial fluid, both assessed after a single administration. The combined MSU/LPS dosing of 2 mg/5 μg gave the most robust response.

**
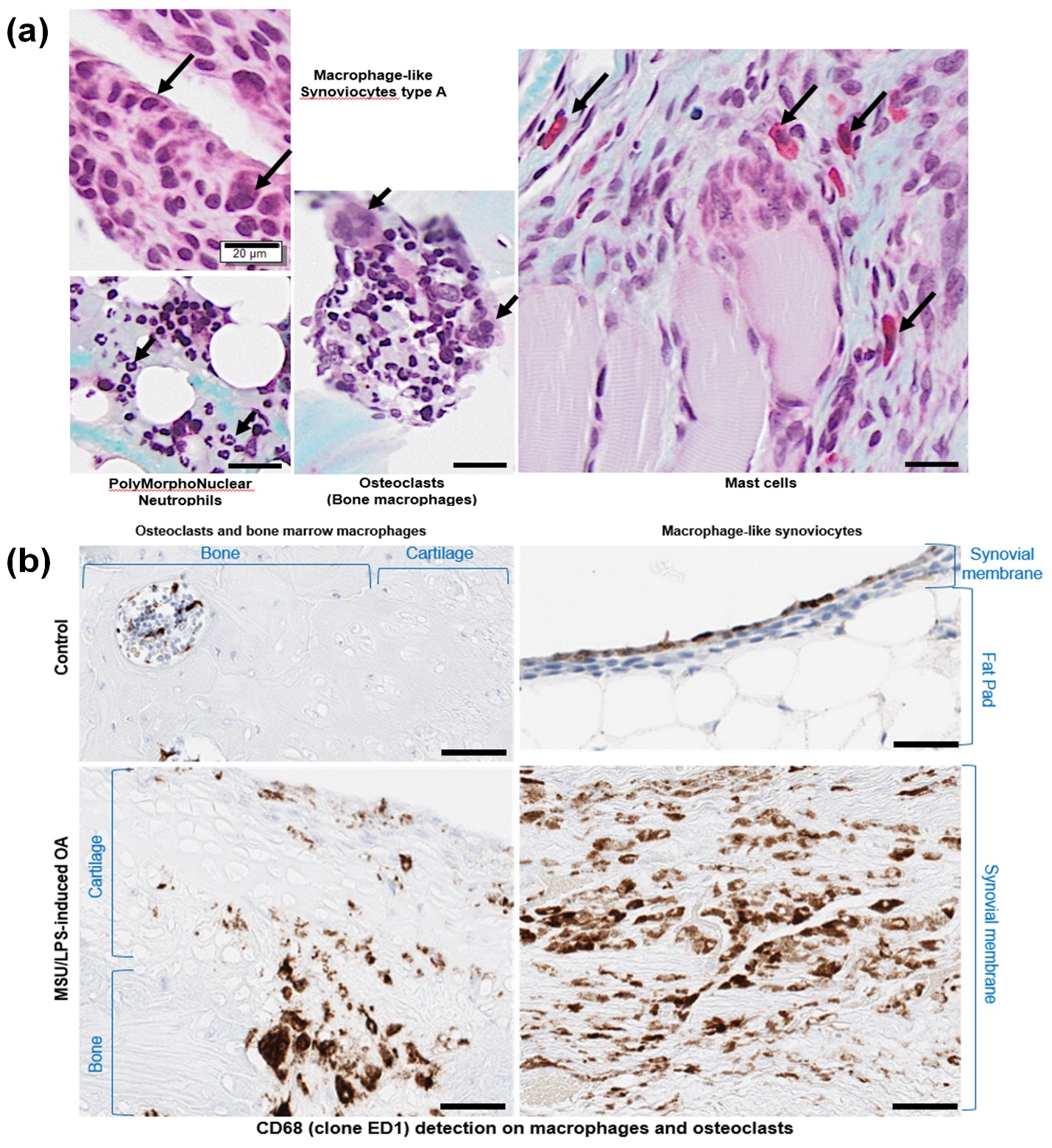
**

**Supplementary figure 2 -** Cell types identified in MSU/LPS-injected joints at day 70 (day 14 after the fifth MSU/LPS injection). (a) Main cell types identified in MSU/LPS-injected joints were polymorphonuclear neutrophils, mast cells and macrophages. Scale bars represent 20 µm. (b) Immunohistochemistry of CD68 confirmed the presence of macrophages and osteoclasts. Scale bars represent 50 µm.

**

**

**Supplementary figure 3 -** Surface reconstructions of three-dimensional micro-CT scans from the rat knee joint. Reconstructions from images from five rats, obtained at day 69 (day 13 after the fifth administration of MSU/LPS). No signs of osteophytes could be detected in these reconstructions.
